# Cellular Mechanisms of Multiple Myeloma Bone Disease

**DOI:** 10.1155/2013/289458

**Published:** 2013-05-29

**Authors:** Angela Oranger, Claudia Carbone, Maddalena Izzo, Maria Grano

**Affiliations:** Department of Basic Medical Sciences, Neurosciences and Sense Organs, Section of Human Anatomy and Histology, University of Bari, Piazza Giulio Cesare 11, 70124 Bari, Italy

## Abstract

Multiple myeloma (MM) is a hematologic malignancy of differentiated plasma cells that accumulates and proliferates in the bone marrow. MM patients often develop bone disease that results in severe bone pain, osteolytic lesions, and pathologic fractures. These skeletal complications have not only a negative impact on quality of life but also a possible effect in overall survival. MM osteolytic bone lesions arise from the altered bone remodeling due to both increased osteoclast activation and decreased osteoblast differentiation. A dysregulated production of numerous cytokines that can contribute to the uncoupling of bone cell activity is well documented in the bone marrow microenvironment of MM patients. These molecules are produced not only by malignant plasma cells, that directly contribute to MM bone disease, but also by bone, immune, and stromal cells interacting with each other in the bone microenvironment. This review focuses on the current knowledge of MM bone disease biology, with particular regard on the role of bone and immune cells in producing cytokines critical for malignant plasma cell proliferation as well as in osteolysis development. Therefore, the understanding of MM pathogenesis could be useful to the discovery of novel agents that will be able to both restore bone remodelling and reduce tumor burden.

## 1. Introduction

Multiple myeloma (MM) is a hematologic malignancy characterized by the accumulation of monoclonal plasma cells (over 10% by definition) in the bone marrow (BM) [1], the presence of monoclonal immunoglobulin (Ig) in the serum or urine, osteolytic bone lesions, renal disease, and immunodeficiency. It is mainly a disease of old patients, with a median age at diagnosis of 65–70 years. In almost all cases, MM is preceded by a premalignant disease well known as monoclonal gammopathy of undetermined significance (MGUS) [2, 3], that affects 2% of the population above the age of 50. Both genetic and environmental factors have been implicated in MGUS progression to MM [4], but the reasons why it happens in only a small proportion of patients are yet unclear. Progression to MM is correlated with changes in the BM microenvironment, including increased angiogenesis, suppression of the immune response, and increased bone resorption [5]. More than 80% of MM patients develop osteolytic bone disease, often associated with hypercalcemia and skeletal-related events such as severe bone pain, vertebral compression fractures, and pathologic fractures. Importantly, pathologic fractures affect 40% to 50% of MM patients, increasing the risk of death by more than 20% compared with patients without fractures [6, 7]. Thus, osteolytic lesions have a negative impact on both quality of life and survival of patients. 

It was well documented that the interaction of malignant plasma cells with BM stromal cells (BMSCs) is crucial for the homing and growth of malignant plasma cells as well as for the impairment of osteoclast (OC), the bone resorbing cell, and osteoblast (OB), the bone forming cell, activities. In particular, in areas adjacent to myeloma cells, OC activity increases, resulting in enhanced bone resorption, and OB activity declines with consequent reduced bone formation [8]. Therefore, bone remodeling, in which OC and OB activities are tightly coupled, is disrupted in MM. 

It was also demonstrated that several factors produced as a result of MM cell—BMSC interactions also alter the functions of the host immune cells, thus interfering with immune surveillance, preventing immune mediated tumor rejection [9], and contributing to the MM worsening.

Here, we discuss the pathogenesis of MM bone disease and focus on advances in our understanding of its biology, with particular regard on the role of bone and immune cells in producing cytokines critical for the induction of osteolysis development in MM.

## 2. The Biology of MM Bone Disease

The cross-talk between cells located in the BM microenvironment and bone cells is tightly regulated. Many components of the bone microenvironment are responsible for the proliferation of tumor cells [10–12], that, in turn, promote the formation of a permissive microenvironment for their survival [13–15]. The BM microenvironment refers to both cells located in the BM (malignant plasma cells, stromal and immune cells) and noncellular components, the extracellular matrix (ECM), composed of proteins such as collagen, laminin, and fibronectin and the extracellular fluid containing cytokines and growth factors. The signaling cascades induced by the cells located in the BM microenvironment as well as by bone cells affect not only the propagation and survival of tumor cells but also the differentiation and activation of OCs and OBs, thus contributing to the development of osteolytic lesions.

## 3. MM Cells

**Figure 1:**
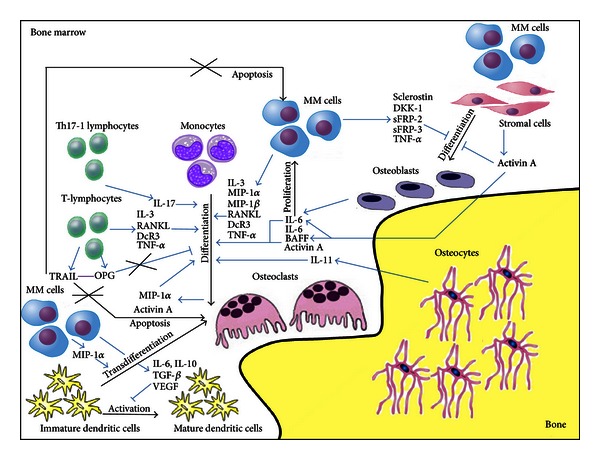
Interaction between bone cells and bone marrow microenvironment cells in promoting both malignant plasma cell survival and bone lesions in MM patients. Myeloma cells can directly support osteoclast formation and activity as well as inhibit osteoblast differentiation by releasing numerous cytokines. Moreover, other molecules can be secreted by bone cells and other cells interacting with each other in the bone microenvironment, thus supporting both the progression of MM tumor burden and the development of MM bone disease.

The BM of patients with MM contains malignant plasma cells that directly, by the production of cytokines, or indirectly, by stimulating BM cell secretion of other factors, contribute to the unbalance between bone resorption and formation, resulting in the development of osteolytic lesions [16]. In fact, bone destruction develops adjacent to MM cells and not in areas of normal BM. In particular, MM cells directly produce factors implicated in both OC activation and OB inhibition. Among the factors implicated in OC activation, it was demonstrated that malignant plasma cells produce decoy receptor 3 (DcR3), interleukin-3 (IL-3), macrophage inflammatory protein-1*α* (MIP-1*α*), macrophage inflammatory protein-1*β* (MIP-1*β*), and tumor necrosis factor-*α* (TNF-*α*) (Figure 1). 

We demonstrated that DcR3, a member of the TNF receptor superfamily and known to be involved in OC differentiation [17], was overexpressed by malignant plasma cells and T-lymphocytes obtained from MM patients with osteolysis [18, 19]. 

Lee et al. demonstrated the ability of MM cells to overexpress another pro-osteoclastogenic factor: IL-3 [20]. Furthermore, they found that BM plasma samples from MM patients stimulated OC formation *in vitro*, and the effect was reversed by the addition of a neutralizing antibody to IL-3 [20]. Other authors reported that IL-3 promotes both the increase of pre-OC number and their fusion into mature OCs [21], thus confirming the potential role of IL-3 as an OC stimulatory factor in MM bone disease. In addition, a contribution of IL-3 in the inhibition of bone formation in MM has also been reported. It inhibits OB differentiation of primary mouse and human stromal cells treated with BMP-2 in a dose-dependent way without affecting cell growth [22]. Thus, IL-3 appears to be a potential mediator of myeloma bone disease, playing a dual role in MM both stimulating OC activity and inhibiting OB differentiation. All these data are in agreement with the IL-3 elevated levels found in BM and blood of patients with MM [23]. 

Another cytokine produced at high levels by MM cells is MIP-1*α* [24]. Its high BM serum levels correlate with osteolytic lesions and survival in MM patients [24]. It is a low molecular weight chemokine which can interact with its receptors, CCR1 and CCR5, expressed by monocytes and BMSCs [25–27]. MIP-1*α* acts as a chemoattractant [28] and has a role in hematopoiesis, OC recruitment, and differentiation in BM [25, 29, 30]. It was demonstrated that in MM bone disease MIP-1*α* induces OC differentiation from monocytes as well as from immature dendritic cells (DC) by transdifferentiation [31]. This reciprocal effect of MIP-1*α* on DC and OC differentiation further contributes to the immunosuppression and bone destruction in MM. MIP-1*α* also induces survival, growth, and chemotaxis of MM cells [32]. The dual activity of MIP-1*α* has been targeted *in vivo* with different strategies. In a mouse model of MM bone disease, it was shown that both antisense sequence and neutralizing antibody against MIP-1*α* restored bone remodelling and inhibited tumor growth [33, 34]. Moreover, the inhibition of CCR1 was associated with impairment of osteoclastogenesis and OC-induced tumor cell proliferation *in vitro*, suggesting that the MIP-1*α*/CCR1 pathway is an important target in MM bone disease [35]. 

MIP-1*β* is a highly homologous chemokine of MIP-1*α* constitutively secreted by MM cells, that similarly to MIP-1*α* induces the development of osteolytic bone lesions [36]. 

About the possibility that MM cells produce also receptor activator of NF-**κ**B ligand (RANKL), a well known pro-osteoclastogenic molecule; literature data are still controversial. RANKL binds its receptor, RANK, expressed by OC precursors, and induces OC formation in the presence of Macrophage colony-stimulating factor (M-CSF) [37]. Alternatively, RANKL could interact with osteoprotegerin (OPG), a secreted member of the TNF receptor superfamily, that, by binding to and blocking the effect of RANKL [38], inhibits OC formation. Thus, the ratio of OPG/RANKL is crucial for OC development, and its unbalance is associated with bone disease [39]. Several studies conducted on human MM cells from patients [40–42], human MM cell lines, and a murine MM cell line [43] reported RANKL expression by myeloma cells. On the contrary, other studies were not able to detect RANKL expression in human myeloma cell lines or primary myeloma cells [44–48]. Independently on the possibility that MM cells could produce RANKL, it was well documented that its overexpression in BM microenvironment correlated with BMSCs and T-lymphocytes production, as will be discussed. These data were in agreement with the high BM plasma levels of RANKL found in MM patients [45] and with the high circulating RANKL serum levels demonstrated by Jakob et al. [49]. In particular, these last researchers found that serum total-RANKL reflects advanced disease, lytic bone destruction, and poor prognosis in MM [49]. 

Malignant plasma cells not only produce cytokines involved in OC survival and formation but also secrete molecules responsible for the inhibition of OB activity. It was demonstrated that MM cells secrete soluble frizzled-related proteins-2 and -3 (sFRP-2 and -3) [50–52], Dickkopf-1 (DKK-1) [53], and sclerostin [54] (Figure 1), all implicated in the inhibition of the canonical wingless-type (Wnt) signaling. The canonical Wnt pathway is one of the most relevant signaling regulating OB differentiation. Wnts are secreted cysteine-rich glycoproteins known as regulators of hematopoietic and mesenchymal cell differentiation as well as of embryonic development [55–57]. The activation of canonical Wnt signaling, induced by binding of Wnt proteins to both Frizzled receptor and low-density lipoprotein receptor-related protein (LRP-5/6) coreceptor, is followed by *β*-catenin translocation into the nucleus, [58, 59] resulting in the activation of major OB transcription factors. Thus, the presence, in the bone microenvironment, of secreted antagonists, such as sFRPs which interfere with Wnt/Frizzled receptor binding, or DKK proteins and sclerostin, which bind the coreceptor LRP5/6 [60], could negatively regulate osteoblastogenesis. In particular, sFRP-2 and -3 have been reported to be produced both by primary MM cells from patients and MM cell lines. It was shown that recombinant sFRP-2 inhibits OB differentiation [50] and that neutralizing sFRP-2 in conditioned media from MM cell lines partially reversed the inhibition of OB differentiation. In addition, it was also demonstrated that sFRP-3 was upregulated in MM patients [51, 52]. Moreover, it was reported that DKK-1, highly expressed in BM of MM patients with osteolytic lesions, is apparently involved in early stages of bone disease [53]. It was implied in the development of MM osteolytic lesions because of both its inhibitory effect on OB formation and its effect in increasing OC formation through the upregulation of RANKL and inhibition of OPG secretion, by inhibiting Wnt-3A [61]. Similarly, we demonstrated the expression of sclerostin by myeloma cells and the possibility that its contribution in the development of MM bone disease could be related to both a direct induction of OB suppression with reduced bone formation and an indirect activation of OC bone resorption through the unbalanced RANKL/OPG ratio [54–62]. Moreover, Terpos et al. demonstrated that patients with active myeloma have elevated circulating sclerostin levels, which correlate with advanced disease features including severe bone disease [63].

MM cells produce TNF-*α* (Figure 1), a factor that can induce OC formation [64, 65], promote MM cell proliferation by increasing Interleukin-6 (IL-6) production by BMSCs [66], also inhibit mesenchymal stem cell proliferation, and induce mature OB apoptosis [64]. Moreover, it was recently reported that the OB transcriptor factor Runx2 mediates the effects of TNF-*α* on OBs [67]. In particular, they found that the knockdown of Runx2 in mesenchymal stem cells abolished the capacity of TNF-*α* to block proliferation and differentiation of the cells. These results show an important link between Runx2 and TNF-*α*'s capacity to inhibit OB differentiation. It was also demonstrated that MM cells selectively suppress BMSCs differentiation into functional OBs, while adipogenesis is not affected [68–70].

## 4. Bone Marrow Stromal Cells (BMSCs)

MM cells adhere to both BMSCs and ECM into the BM. The adhesion of tumor cells to BMSCs activates many pathways resulting in upregulation of antiapoptotic proteins and cell cycle regulating cytokines [71]. The main cytokines upregulated are RANKL, IL-6, B-cell activating factor (BAFF), and Activin A (Figure 1). 

Specifically, the interaction between MM cells and BMSCs provokes IL-6 secretion in BMSCs via NF-*κ*B-dependent transcription [13, 72]. IL-6 is known to regulate MM cell proliferation and inhibition of both myeloma plasma cell apoptosis [73, 74] and OC differentiation [75].

MM cell adhesion to BMSCs also promotes BAFFproduction via NF-*κ*B activation [76]. BAFF is a member of the TNF protein super family, crucial for the maintenance and homeostasis of normal B-cell development, and has been shown to both confer a survival advantage on MM cells [76–78] and to promote RANKL-independent osteoclastogenesis [79].

Recently, Activin A, a TGF-*β* family member secreted by BMSCs and OCs after MM cells interaction [80], was identified to have a crucial role in the pathogenesis of MM bone disease. Activin A modulates bone remodeling by dual activity as OC promoter and inhibitor of OB differentiation. In MM, high Activin A levels in both BM and peripheral blood are associated with advanced bone disease [80]. Terpos et al. also demonstrated that patients with newly diagnosed symptomatic myeloma had increased circulating Activin A levels compared with controls and that these high levels correlate with advanced features of myeloma [81].

## 5. Osteoclasts (OCs)

OCs are bone resorbing cells whose activity and viability are upregulated in MM bone disease because of the presence, in the BM microenvironment, of several factors implicated in their differentiation and activation (RANKL, IL-3, IL-6, MIP-1*α*, MIP-1*β*, BAFF, DcR3, TNF-*α*, and Activin A). Not only MM, BMSCs, and immune cells but also OCs represent the source of some pro-osteoclastogenic molecules. 

In particular, it was demonstrated that OCs could secrete proteins, such as Activin A [80] and MIP-1*α* [82], implicated in pre-OC requirement and OC differentiation and activation (Figure 1).

## 6. Osteoblasts (OBs)

It has been reported that OBs, the bone forming cells, may contribute to MM pathogenesis by both supporting MM cell growth and survival [83] and contributing to osteolysis development. This could potentially result from the ability of OBs to secrete IL-6 in coculture system with myeloma plasma cells, therefore, inducing MM cell growth (Figure 1). Other mechanisms include the possible role of OBs in stimulating MM cell survival by blocking MM cell apoptosis mediated by TNF-related apoptosis-inducing ligand (TRAIL), through OPG release, a receptor for both TRAIL and RANKL [84]. Thus, the suppression of OB activity is responsible for both bone destruction and progression of myeloma tumor burden. It was previously described that MM cells secreted several Wnt antagonists that are responsible for suppression of OB differentiation and activity in MM such as DKK-1 [53], sFRP-2 [50], sFRP-3 [52], and sclerostin [54]. Moreover DKK-1 and sclerostin also disrupt Wnt-regulated OPG and RANKL production by OBs, thus contributing to the bone destruction in MM patients acting not only on OB inhibition but also on OC over-activation. Consistently, all these factors are significantly overexpressed in patients with MM who present lytic bone lesions. Studies have shown that blocking DKK-1 and activating Wnt signaling prevent bone disease in MM but are also associated with a reduction in tumor burden [85–87].

## 7. Osteocytes

Osteocytes, the bone cells entrapped into the mineralized bone matrix, regulate bone remodelling at least partially, as a result of their cell death triggering OC recruitment. It was recently demonstrated that the number of viable osteocytes was significantly smaller in MM patients with bone lesions than in those without them or in healthy controls and negatively correlated with the number of OCs [88]. The authors also showed that MM cells cause an upregulation of osteocyte production of the pro-osteoclastogenic cytokine interleukin-11 (IL-11) (Figure 1) and that its expression was higher in the MM patients with bone lesions than in those without them [88]. Thus among the bone cells, not only OCs and OBs but also osteocytes are involved in MM-induced OC formation.

## 8. T-Lymphocytes

T cells are immune cells that could regulate OC and OB formation, lifespan, and activity [89, 90]. Thus, they could contribute to bone remodeling in both health and disease by producing specific proteins. In the peripheral blood of MM patients, the absolute count of lymphocytes and T cells is often deficient because of a reduction in the number of CD4+ T cells, associated with a significantly decreased ratio of CD4/CD8 T cells, particularly evident in patients with progressive disease [91–93]. Recently, a subclass of CD4+ cells, named regulatory T cells (Tregs), has been identified in MM [94]. Tregs are cells involved in the control of self-tolerance and immune homeostasis, with suppressive capabilities. These cells are early induced during tumor development and are shown to contribute to tumor tolerance [95, 96]. The presence of Tregs in tumors is associated with a poor prognosis [97]. Patients with many different types of cancers had increased number of Tregs in their blood, tumor mass, and draining lymph nodes [98, 99]. Conflicting reports have been published on the frequency of Treg cells in MM patients, with studies showing either their decrease or increase [100–102]. In particular, Prabhala et al. demonstrated that the Tregs were significantly reduced in MGUS and MM subjects [103]; other authors demonstrated that Treg cells were expanded only in patients with MM at diagnosis, but not in those in remission or in patients with MGUS. Another study involving MM patients and MGUS subjects showed that in both MGUS and untreated MM subjects, as well as treated MM patients, the frequency of Treg cells was increased compared with healthy controls [100].

In MM, abnormalities within T-cell compartment have also been reported in BM, in which T-cell count increased, and, differently from the peripheral blood, a slightly increased CD4/CD8 ratio was observed. In BM from patients with MM, Dhodapkar et al. demonstrated a high proportion of a distinct lineage of T helper cells producing interleukin 17 (IL-17), called Th17-1 cells [104]. IL-17 is a cytokine that, in addition to exerting an effect on cell survival [105], has also been identified as a key mediator of bone disease in MM [106]. Interestingly, the extent of lytic bone disease appears to be largely mediated by IL-17 produced by Th17-1 cells, independently of the tumor burden, underscoring the crucial interplay of the immune system with the tumor microenvironment in the pathogenesis of MM [106]. 

MM-activated T cells have the capacity to secrete a wide variety of pro-osteoclastogenic cytokines that become critical in the induction of osteolysis development in MM. In particular, it was demonstrated that MM T cells produce high levels of IL-3 [107], RANKL [19], DcR3 [19], and TNF-*α* [19], all involved in OC formation and activation (Figure 1). We demonstrated that T cells from MM bone disease patients also express high levels of TRAIL, known to be a proapoptotic molecule, and the antiosteoclastogenic protein OPG [44]. We showed that the OPG/TRAIL interaction could contribute to the elevated formation of long lifespan OCs in MM patients [44] (Figure 1).

It was also demonstrated the presence of a vicious loop that involved molecules produced by MM cells, T-lymphocytes, and BMSCs [108]. In particular Giuliani et al. showed that MM cells, by secreting interleukin-7 (IL-7), are able to induce an upregulation of RANKL and a downregulation of interferon-*γ* (inhibitor of OC formation) secretion by T-lymphocytes [109]. Other authors demonstrated that IL-7 stimulates IL-6 secretion by BMSCs [110]. High levels of IL-6 in the BM environment could induce IL-7 production by MM cells, which in turn contribute to maintain high IL-6 levels and stimulate RANKL expression by T cells. In addition, it has been shown that IL-7 can also contribute to the development of osteolytic lesions in MM by inhibiting the differentiation of OBs. In fact, the usage of IL-7 blocking antibodies partially blunts the inhibitory effects of MM cells on OB differentiation [111]. 

## 9. Dendritic Cells (DCs)

DCs are specialized antigen-presenting cells able to initiate immune responses [112]. DCs derive from myeloid or lymphoid progenitors, and their functions are determined by their origin as well as by their maturation stage, which depends on the signals received from pathogens and T cells. In mice, MM cells or tumor culture-conditioning medium inhibit the differentiation and activation of DCs, as shown by the lower expression of DC-related antigens and compromised capacity to activate allospecific T cells [113]. It was documented that circulating DCs from MM patients were dysfunctional because they failed to upregulate costimulatory molecules required for activation [114]. It was suggested that a reduced function of DCs indicates the progression of the disease [114]. Cytokines actively produced by myeloma cells such as IL-6, IL-10, transforming growth factor-*β* (TGF-*β*), and vascular endothelial growth factor (VEGF) [114], abundant in the BM as well as in the serum [115], play a role in preventing the development of functional DCs (Figure 1). Furthermore, DCs from MM patients have reduced phagocytic capacity [116]. In addition, monocyte-derived DCs exhibit downregulated expression of activation markers and impaired presentation capacity to T cells [115]. Impaired activity of DCs may be also linked to the upregulation of Tregs [117], consistently with the observation of some authors that found an increase of Treg number in MM subjects. 

## 10. Novel Antimyeloma-Related Bone Disease Drugs

Current pharmacological strategies in MM have resulted in improved patient overall survival, but no definitive treatment has been as yet achieved. Nowadays, consisting with the improved survival of MM patients, treatment of bone disease has assumed high relevance. Until recently, therapeutic cures for MM bone disease, aimed at reducing the development of new osteolytic lesions, included bisphosphonates, radiotherapy and surgery. Several promising preclinical studies including novel bone-targeted agents suggest that restoring bone homeostasis may lead to inhibition of both bone pain and tumor growth. Here, the current bone-directed drugs are described, with particular regard to their mode of action and targets (Table 1).

*Denosumab*. Denosumab is a RANKL-neutralizing antibody (AMG165), successfully used in MM patients to inhibit bone resorption markers. A single subcutaneous administration of denosumab induces an important inhibition of bone resorption markers. A randomized clinical trial showed that denosumab inhibits bone resorption and prevents fracture development even in MM patients refractory to bisphosphonates therapy [118]. Recently, it was also demonstrated that RANKL inhibition with denosumab is as efficacious as zoledronic acid in terms of decreasing fracture development. Denosumab is a well-tolerated drug. Asthenia and peripheral edema represent the only side effects demonstrated on patients that assume the drug [119]. Currently, denosumab continues to remain in clinical development for MM.

*Anti-BAFF—Neutralizing Antibody*. BAFF is an MM growth factor produced by OC and BMSC that mediates both MM cells-BMSC adhesion and MM cell survival [76, 120]. It was demonstrated that *in vivo* neutralizing antibodies against BAFF (LY2127399) significantly reduce OC differentiation and inhibit tumor burden [121]. Currently a clinical trial combining BAFF-neutralizing antibody with bortezomib, a proteasome and NF-*κ*B signaling pathway inhibitor, is ongoing.

*CCR1-Inhibitors*. The MIP-1*α*/CCR1 pathway is involved in OC differentiation and promotes MM cell survival, making it a possible therapeutic target. *In vitro* and *in vivo* studies showed that inhibition of MIP-1*α* by antisense strategies prevents the development of osteolytic lesions and inhibits tumor growth [33]. Similar results have been shown with MLN3897, a specific orally available CCR1 inhibitor. This drug inhibits both OC formation and MM cell proliferation [122]. Further clinical trials on patients with MM bone disease will be needed to confirm these interesting preliminary data.

*DKK-1 Antagonists*. It is well known that the Wnt inhibitor DKK-1 plays a key role in mediating OB inhibition in MM [123]. Thus, numerous strategies to block DKK-1 activity have been developed. *In vitro* assays show that DKK-1 inhibition via a specific neutralizing antibody promote OB differentiation and function [86, 124]. Moreover, *in vivo* studies using DKK-1 inhibitors on murine and humanized models of MM-induced bone disease show increased OB number and bone formation, thus resulting in osteolytic lesion improvement [87, 125]. Moreover, blocking DKK-1 also resulted in reduction of tumor growth [124]. Currently, phase 1 clinical trials are ongoing combining DKK-1-neutralizing antibody and bisphosphonates. In particular, BHQ880, an anti-DKK-1 monoclonal antibody, in combination with zoledronic acid is being studied. 

*Activin A Antagonists*. Activin A, a cytokine upregulated in MM patients with extensive bone disease [81], is able to both stimulate OC differentiation and inhibit OB formation. In two myeloma mouse models, the administration of an Activin A chimeric inhibitor (RAP-011) derived from the fusion of the extracellular domain of activin receptor IIA and the constant domain of the murine IgG2a [126] or a soluble Activin A receptor type IIA fusion protein (ActRIIA.muFc) blocks the development of osteolytic bone lesions by both inhibiting OC development and stimulating osteoblastogenesis [80, 127]. Moreover, RAP-011 effectively reduced tumor growth [80]. It was just demonstrated that the humanized counterpart of RAP-011, sotatercept (ACE-011), stimulates bone formation and inhibits bone resorption markers in postmenopausal women. Thus, the inhibition of Activin A may be a promising approach for the treatment of myeloma-related bone lesions. Ongoing clinical trials are evaluating sotatercept role in MM.

## 11. Conclusions

Recently, a lot of studies demonstrated a close relationship between the immune and skeletal systems as well as tumor growth and bone cell activity in MM bone disease. Nowadays, it is evident that not only MM cells but also bone cells, BMSCs, and immune cells are critical players in the pathogenesis of MM bone disease, thus contributing to the development of osteolysis. These cells as well as their products participate in both OC development and OB inhibition leading to bone destruction in MM. In the BM microenvironment, a vicious circle between the bone destructive process and tumor progression that feed each other was maintained. Thus, the inhibition of bone resorption could decrease both myeloma bone disease and tumor progression. The discovery of novel agents with dual activity on bone remodelling may also result in improvement of bone disease besides prevention of osteolytic lesions. Therefore, agents restoring bone balance in MM represent a novel strategy to overcome osteolytic disease and MM tumor growth.

## Figures and Tables

**Table 1:** Novel drugs for multiple myeloma bone disease.

Name of the drug	Action	Bone target cell/s
Denosumab	RANKL neutralizing antibody	OCs
LY2127399	BAFF neutralizing antibody	OCs
MLN3897	CCR1 inhibitor	OCs
BHQ880	DKK1 neutralizing antibody	OBs
ACE-011 (sotatercept)	Activin A neutralizing receptor	OCs and OBs
